# Integrated Cross-Sectional Multiplex Serosurveillance of IgG Antibody Responses to Parasitic Diseases and Vaccines in Coastal Kenya

**DOI:** 10.4269/ajtmh.19-0365

**Published:** 2019-11-25

**Authors:** Sammy M. Njenga, Henry M. Kanyi, Benjamin F. Arnold, Sultani H. Matendechero, Joyce K. Onsongo, Kimberly Y. Won, Jeffrey W. Priest

**Affiliations:** 1 Eastern and Southern Africa Centre of International Parasite Control, Kenya Medical Research Institute, Nairobi, Kenya;; 2 Francis I. Proctor Foundation, University of California, San Francisco, California;; 3 Department of Preventive and Promotive Services, Neglected Tropical Diseases Programme, Ministry of Health, Nairobi, Kenya;; 4 World Health Organization Country Office, Nairobi, Kenya;; 5 Division of Parasitic Diseases and Malaria, Centers for Disease Control and Prevention, Atlanta, Georgia;; 6 Division of Foodborne, Waterborne and Environmental Diseases, Centers for Disease Control and Prevention, Atlanta, Georgia

## Abstract

Accurate and cost-effective identification of areas where co-endemic infections occur would enable public health managers to identify opportunities for implementation of integrated control programs. Dried blood spots collected during cross-sectional lymphatic filariasis surveys in coastal Kenya were used for exploratory integrated detection of IgG antibodies against antigens from several parasitic infections (*Wuchereria bancrofti*, *Schistosoma mansoni*, *Plasmodium* spp., *Ascaris lumbricoides*, and *Strongyloides stercoralis*) as well as for detection of responses to immunizing agents used against vaccine-preventable diseases (VPDs) (measles, diphtheria, and tetanus) using a multiplex bead assay (MBA) platform. High heterogeneity was observed in antibody responses by pathogen and antigen across the sentinel sites. Antibody seroprevalence against filarial antigens were generally higher in Ndau Island (*P* < 0.0001), which also had the highest prevalence of filarial antigenemia compared with other communities. Antibody responses to the *Plasmodium* species antigens circumsporozoite protein (CSP) and merozoite surface protein-1 (MSP-1)_19_ were higher in Kilifi and Kwale counties, with Jaribuni community showing higher overall mean seroprevalence (*P* < 0.0001). Kimorigo community in Taita–Taveta County was the only area where antibody responses against *S. mansoni* Sm25 recombinant antigen were detected. Seroprevalence rates to *Strongyloides* antigen NIE ranged between 3% and 26%, and there was high heterogeneity in immune responses against an *Ascaris* antigen among the study communities. Differences were observed between communities in terms of seroprevalence to VPDs. Seroprotection to tetanus was generally lower in Kwale County than in other counties. This study has demonstrated that MBA holds promise for rapid integrated monitoring of trends of infections of public health importance in endemic areas.

## INTRODUCTION

Several major infectious diseases occur in sub-Saharan Africa including malaria and neglected tropical diseases (NTDs), which are particularly common among resource-poor populations.^1–3^ Consequently, several of these diseases are co-endemic and past studies in the region have identified subgroups that are polyparasitized with soil-transmitted helminth (STH) infections, filarial parasites, and malaria.^4–6^ Lymphatic filariasis (LF) caused by *Wuchereria bancrofti* is principally confined to the coastal region of Kenya where ecological factors are suitable for its transmission^7^; the disease co-occurs with other infectious diseases such as STH infections, schistosomiasis, lower respiratory infections, and malaria.^8–10^

In the past, lack of resources often compounded by competing health priorities in sub-Saharan Africa has led to insufficient commitments to control NTDs. More recently, however, implementation of successful public–private partnerships for health have availed resources for control and/or elimination of NTDs as public health problems. In 2000, the WHO Global Programme to Eliminate Lymphatic Filariasis (GPELF), launched in response to World Health Assembly resolution WHA50.29, urged member states to initiate activities to eliminate LF as a public health problem, a goal subsequently targeted for 2020.^11^ Community-wide mass drug administration (MDA) of antifilarial drugs for 4–6 years is recommended for LF elimination, and modeling studies have estimated adequate treatment coverage to be at least 65% of total population in endemic areas.^12,13^ Substantial progress has been made toward elimination of LF, with Togo being the first country in sub-Saharan Africa to be recognized by the WHO for eliminating the disease as a public health problem.^14,15^ The Kenyan Ministry of Health launched an LF elimination program in 2002, but the program did not sustain MDA campaigns annually as per GPELF recommendations.^16,17^ In 2015, the Ministry of Health successfully appealed to the WHO Regional Office for Africa and other partners for support to reestablish annual MDA campaigns. Subsequently, the WHO Country Office in Nairobi, Kenya, selected the Eastern and Southern Africa Centre of International Parasitic Control (ESACIPAC), which is part of the Kenya Medical Research Institute (KEMRI), to conduct a comprehensive epidemiological assessment of LF infection before restarting MDA.

Antibody levels can provide valuable information about exposure to infections and, thus, can be helpful for characterizing pathogen transmission dynamics.^18^ Because parasite antigens are generally known to elicit an IgG response that can be detected for a long period of time, serological analysis of young children could provide an estimate of more recent exposure.^19,20^ A state-of-the-art multiplex bead assay (MBA) serological platform that enables simultaneous detection of antibodies against multiple antigens using a small volume of blood sample dried on filter paper (10 µL dried blood spots [DBS]) has been developed as a tool for integrated biomarker surveys.^21–23^ The MBA has successfully been used to simultaneously measure antibody responses to multiple parasitic diseases of public health importance as part of a vaccine-preventable disease (VPD) serological survey in Cambodia.^24^ The platform has also been used to simultaneously assess IgG responses to a panel of malaria antigens.^25,26^ In the present study, the MBA platform was used for multiplex serosurveillance of diseases of public health importance by testing for antibodies against LF and several other parasitic diseases (malaria, schistosomiasis, ascariasis, and strongyloidiasis) as well as seroprevalence to selected VPDs (measles, diphtheria, and tetanus).

## MATERIALS AND METHODS

### Study design and samples.

The DBS samples used in this study were collected during cross-sectional LF surveys conducted in October 2015 in 10 sentinel sites located across the coastal region in Taita–Taveta, Kwale, Kilifi, Tana River, and Lamu counties. Counties are devolved subnational governments which relate with the national government as distinct administrative units. In this study, a sentinel site is defined as a rural community (village) from which data collection was conducted with the intention of follow-up testing for monitoring and evaluation of the LF elimination program. A detailed description of the 10 sentinel sites and characteristics of the study participants were provided in an earlier report.^17^ Briefly, 300 persons aged 2 years or older in each sentinel site were targeted for the LF survey as recommended in the WHO guidelines.^11^ The middle finger of consenting individuals was cleaned using a cotton ball soaked in 70% isopropyl alcohol. After drying, the tip of the finger was pricked using a sterile lancet and blood was collected into capillary tubes for detection of circulating filarial antigen (CFA) by immunochromatographic card test (ICT). Additional blood sample from the same prick site was collected onto a filter paper with six extensions, each calibrated to absorb 10 µL volume (TropBio Pty Ltd., Queensland, Australia), and then allowed to air-dry for ≥ 4 hours.

### Recombinant antigens and coupling to microsphere beads.

Recombinant *Schistosoma mansoni* glutathione-*S*-transferase (GST) protein was expressed from pGEX 4T-2 plasmid (GE Healthcare, Piscataway, NJ) and purified as previously described.^27^ Glutathione-*S*-transferase fusion proteins that included protein sequences from *Brugia malayi* (Bm33^22^ and Bm14^20^), *Strongyloides stercoralis* (NIE^28^), and *Plasmodium falciparum* 3D7 strain (MSP1_19_^29^) were expressed and purified as previously described. A *W. bancrofti* Wb123-GST fusion protein was a kind gift from T. Nutman (NIH, Bethesda, MD). These proteins were coupled to SeroMap beads (Luminex Corp., Austin, TX) using the protein quantities and buffer conditions previously described.^24^ *Schistosoma mansoni*–native soluble egg antigen (SEA) was a kind gift of E. Secor (CDC, Atlanta, GA), and recombinant *S. mansoni* Sm25 antigen was expressed using the Baculovirus system previously described.^30^ Both proteins were coupled to SeroMap beads using the protein quantities and buffer conditions previously described.^30^

Tetanus toxoid (Massachusetts Biological Laboratories, Boston, MA), diphtheria toxoid from *Corynebacterium diphtheriae* (List Biological Laboratories, Campbell, CA), and recombinant measles nucleoprotein (MV-N, Meridian Life Sciences, Memphis, TN)^31^ were purchased from commercial sources. Tetanus toxoid was coupled to SeroMap beads as previously described.^32^ Diphtheria toxoid was coupled in buffer containing 50 mM 2-(N-morpholinoethanesulfonic acid (MES) at pH 5.0 with 0.85% NaCl at a concentration of 60 µg of protein per 1.25 × 10^7^ beads in 1 mL final volume. To decrease background reactivity, measles MV-N was purified by chromatography on a MonoQ HR 5/5 strong anion exchange column (GE Healthcare) before use. Protein (0.75 mg) was loaded onto the column at a flow rate of 1 mL/minute and washed with 4 mL of 25 mM Tris buffer at pH 8.0. This was followed by a 10 mL linear gradient to 0.25 M NaCl in Tris buffer, and then by a 5 mL linear gradient to 1 M NaCl in Tris buffer. Most antibody-reactive MV-N eluted in the high salt fractions between 0.4 and 0.7 M NaCl. These fractions were pooled, concentrated using a Centricon-30 centrifugal filter device (Millipore Corporation, Bedford, MA), and exchanged into a buffer containing 10 mM sodium phosphate with 0.85% NaCl at pH 7.2 (phosphate-buffered saline [PBS]). Approximately 115 µg of protein was recovered (BCA micro assay, Pierce, Rockford, IL). MonoQ purified MV-N was coupled in a buffer containing 50 mM MES at pH 5.0 with 0.85% NaCl at a concentration of 6 µg of protein per 1.25 × 10^7^ beads in 1 mL final volume.

Cloning of the *Plasmodium malariae* MSP1_19_ coding sequence from China I parasite strain is described elsewhere.^33^ This antigen was coupled to 1.25 × 10^7^ SeroMap beads in 50 mM MES buffer at pH 5.0 with 0.85% NaCl at a concentration of 30 µg/mL. The glutaraldehyde protocol of Benitez et al.^34^ was used to cross-link a synthetic 20 amino acid peptide ([NANP]_5_-amide) corresponding to the carboxy-terminal repeat of the *P. falciparum* circumsporozoite protein^35,36^ to purified GST protein. Bead coupling conditions for this antigen were identical to those described previously for the *P. malariae* MSP1_19_ protein.

Purified native hemoglobin (Hb) from *Ascaris suum* worms was a kind gift from P. Geldhof (Ghent University, Gent, Belgium).^37,38^ This antigen was coupled to 1.25 × 10^7^ SeroMap beads in a PBS buffer (pH 7.2) at a concentration of 120 µg/mL.

### Multiplex bead assay.

One bloodspot from each participant, corresponding to about 10 µL of whole blood, was eluted overnight at 4°C with 200 mL of PBS containing 0.05% Tween-20 and 0.05% sodium azide (1:40 serum dilution assuming a 50% hematocrit). A further dilution of 50 mL of eluate into 450 µL of PBS containing 0.5% casein, 0.3% Tween-20, 0.02% sodium azide, 0.5% polyvinyl alcohol, and 0.8% polyvinylpyrrolidone (designated as PBN1) with 3 µg/mL *Escherichia coli* extract was made for a final serum dilution of 1:400. Serum dilutions were centrifuged at a maximum speed to pellet any *E. coli* extract particulates immediately before use. Bloodspot dilutions were assayed in duplicate with antigen-coupled microsphere beads using a Bio-Plex 200 instrument equipped with Bio-Plex Manager 6.1 software (Bio-Rad, Hercules, CA), as previously described.^22,24,26^ The average of the median fluorescent intensity values from the duplicate wells *minus* the background fluorescence from the buffer-only blank was reported as the “median fluorescence intensity *minus* background” (MFI-bg). Samples having a coefficient of variation of > 15% for ≥ 2 positive responses between the duplicate wells were repeated.

### Cutoff determinations.

The WHO International Standard reference sera for tetanus (TE-3; 120 IU/mL) and diphtheria (10/262; 2 IU/mL) purchased from the National Institute for Biological Standards and Control (NIBSC) (Potters Bar, Hertfordshire, United Kingdom) were used to identify MFI-bg cutoff values corresponding to immunoprotection. A tetanus TE-3 value of 10 mIU/mL^39,40^ corresponded to a tetanus toxoid MBA response of 118 MFI-bg units. A diphtheria toxoid MBA response of 4393 MFI-bg units corresponded to the 0.1 IU/mL threshold for complete protection,^41^ and an MBA response of 183 MFI-bg units corresponded to the 0.01 IU/mL threshold for partial protection. Other studies have shown good concordance between the MBA and “gold standard” formats for tetanus and diphtheria.^32,42^ Although a WHO reference standard is available for the quantitation of measles virus–neutralizing antibody responses using the whole virus Plaque Reduction Neutralization Test (PRNT) (NIBSC 97/648; 3 IU/mL), the standard has not been calibrated for use in ELISA format assays^43^ and our MBA only detects IgG antibodies to the measles MV-N protein. In an independent work using the specific bead set from this study, Coughlin et al. (in preparation) determined that receiver operator characteristic (ROC)-optimized MFI-bg cutoff value of 178 MFI-bg units provided good sensitivity and specificity compared with the “gold standard” PRNT.

Multiplex bead assay cutoff estimates for the *S. stercoralis* NIE assay and for the three LF antigens (Bm33, Bm14, and Wb123) were assigned using a panel of 94 presumed negative sera donated by anonymous adult U.S. citizens with no history of foreign travel. Test values greater than the mean *plus* three SDs of the presumed negative sample values were considered to be positive. For the *P. malariae* and *P. falciparum* MSP1_19_ assays, log-transformed data were used for the mean *plus* three SD calculation, and the panel used for the *P. falciparum* cutoff included only 65 of the original 94 U.S. adult volunteers. An ROC curve using sera from 41 stool-confirmed, anonymous ascariasis patients, 65 of the adult U.S. citizen volunteers, and sera from 45 anonymous U.S. children was used to identify the cutoff for the *Ascaris* Hb MBA. All of the parasitic disease cutoff values were adjusted to account for differences between the instrument used for cutoff determination at the CDC in Atlanta, GA, and the instrument used to assay the Kenyan sample set at KEMRI in Nairobi, Kenya. Two-fold serial dilutions of the same strong positive sera were assayed on both instruments to generate standard curves for cutoff value adjustment. *Schistosoma mansoni* SEA and Sm25 coupled beads were used in an earlier study, and the adjusted, ROC-assigned cutoff values have been reported elsewhere (965 and 38 MFI-bg units, respectively).^30^

We also estimated seropositivity cutoff points for malaria, LF, and helminth antibody responses using the mean plus three SDs of a seronegative distribution estimated from the study measurements using finite Gaussian mixture models with two components.^44^

### Ethical considerations.

The study received ethical approval from KEMRI Scientific and Ethics Review Unit (SSC No. 3018). In the study villages, chiefs and assistant chiefs arranged for community mobilization meetings during which the purpose of the survey and procedures to be followed were explained. Written informed consent was obtained from every individual who agreed to participate in this study; parents or legal guardians provided signed informed consent forms on behalf of children younger than 18 years of age. The consent information given to the study participants requested for fingerprick blood to test for LF infection and additional DBS specimen for testing of other common diseases in coastal Kenya, including vaccine-preventable diseases. All the DBS samples were transported to Nairobi and analyzed in the KEMRI-ESACIPAC laboratory.

### Statistical analysis.

Mean antibody levels (expressed in MFI-bg units) were analyzed on the log_10_ scale because of skewness in their distribution. We estimated age-dependent mean antibody levels and seroprevalence for each study community using cross-validated, ensemble machine learning, with a library that included the simple mean, linear models, locally weighted regression (loess), and smoothing splines with 2 to 10 degrees of freedom, selected using 10-fold cross-validation.^18^ We estimated age-adjusted geometric mean antibody levels and seroprevalence for each community using targeted maximum likelihood estimation with influence curve-based standard errors.^18^ In cases where seroprevalence approached zero, we estimated exact binomial CIs. Analyses were conducted using R version 3.3.1, and full replication files (data and scripts) are available through the Open Science Framework (https://osf.io/taknp).

## RESULTS

**Figure 1.**
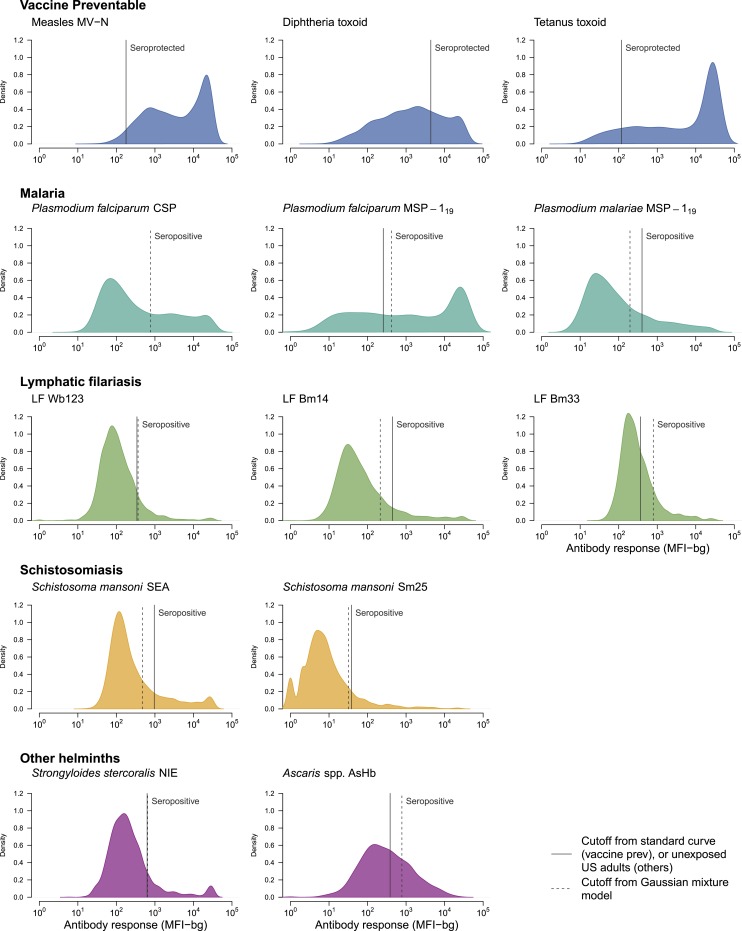
Distribution of IgG antibody levels measured in 10 communities in Kenya’s coastal region, 2015. Antibody response measured in multiplex using median fluorescence intensity minus background (MFI-bg) units on a BioRad Bio-Plex platform. Seroprotection cutoff points for measles, diphtheria, and tetanus estimated using a standard curve from WHO reference standards. Seropositive cutoff points for other antigens estimated using negative control serum samples (solid) and finite Gaussian mixture models (dashed). There was no negative control cutoff point determined for the *Plasmodium falciparum* CSP antigen. Supplemental Table 1 includes cutoff values. Created with script: https://osf.io/d9jrc. This figure appears in color at www.ajtmh.org.

Antibody measurements were obtained from 2,837 individuals (range 271–297 per community) (Supplemental Figure 1). Antibody distributions varied by pathogen and antigen, and seropositivity cutoff values for malaria, LF, and helminth antibody responses derived through ROC curve analysis or mean *plus* three SD calculations were very close to those derived by Gaussian mixture model analysis (Figure 1). We therefore relied on cutoff values derived from the Gaussian mixture model antibody responses for comparability to future studies that may not have access to positive and negative control specimens. Age-dependent patterns and community-level estimates of mean antibody levels and seroprevalence were consistent in their age-dependent patterns and in their community-level ranking (Supplemental Figures 2–6), so we report results based on mean antibody levels in supporting information.

### Antifilarial antibody measurements.

**Figure 2.**
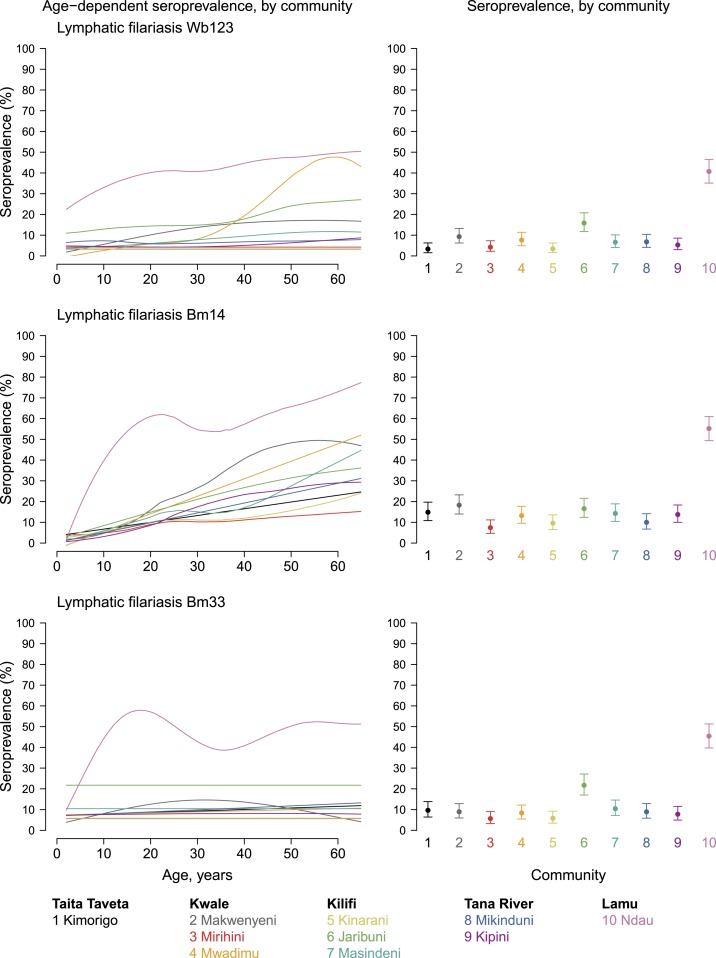
Lymphatic filariasis antibody age-dependent seroprevalence and overall means, stratified by community in Kenya’s coastal region, 2015. Community-level mean seroprevalence is age-adjusted and error bars represent 95% CIs. Supplemental Figure 2 is an extended version of this figure that also includes mean antibody levels. Created with script: https://osf.io/5zkxw. This figure appears in color at www.ajtmh.org.

Individuals who tested positive for LF infection by ICT had higher mean levels of antibody responses against the three recombinant filarial antigens (Supplemental Figure 7). Antibody seroprevalence against all three recombinant filarial antigens were significantly higher in Ndau Island than in other communities, and the difference in seroprevalence in Ndau compared with other communities was markedly greater among persons aged less than 30 years (Figure 2). Antifilarial antibody responses against Bm14 antigen continued to increase with age in all communities. For Wb123, seroprevalence gradually increased with age in Ndau and increased from around the age of 30–35 years in Mwadimu community. Compared with the other communities, Jaribuni had slightly elevated mean antibody responses against Wb123 and Bm33 antigens (*P* < 0.0001), but not for Bm14 antigen (*P* = 0.08). Among the youngest children, quantitative antibody levels differentiated communities more clearly than seroprevalence, owing to high variability in seroprevalence estimates from the small sample sizes in the youngest age strata (Supplemental Figure 8).

### Antibody responses to other parasite antigens.

**Figure 3.**
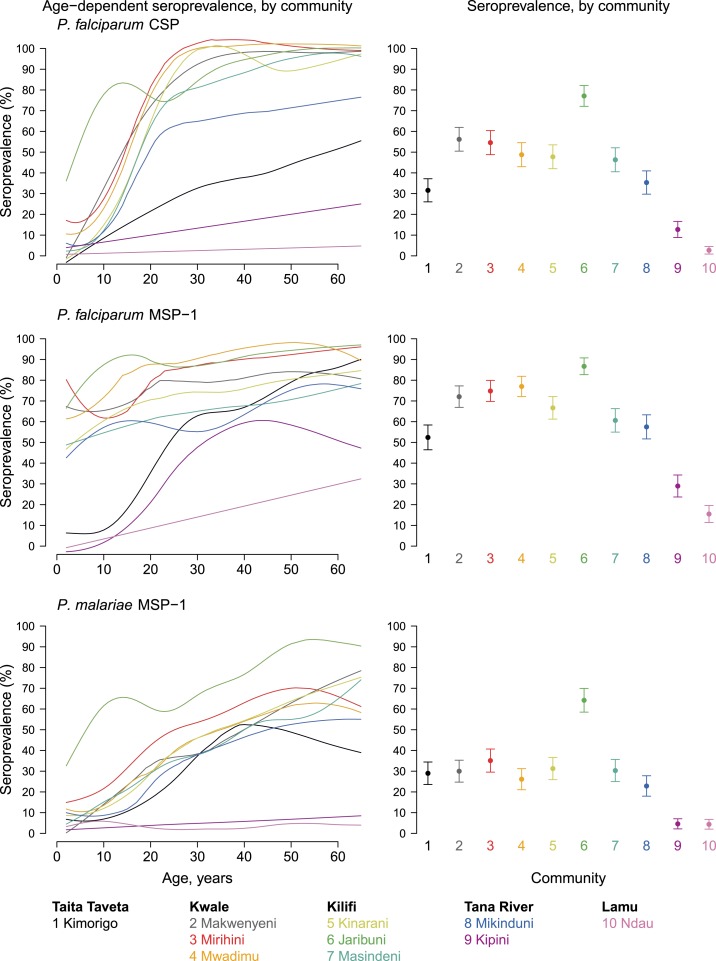
Malarial antibody age-dependent seroprevalence and overall means, stratified by community in Kenya’s coastal region, 2015. Community-level mean seroprevalence is age-adjusted and error bars represent 95% CIs. Supplemental Figure 3 is an extended version of this figure that also includes mean antibody levels. Created with script: https://osf.io/kzfd3. This figure appears in color at www.ajtmh.org.

Antibody responses to the *P. falciparum* CSP and MSP-1_19_ antigens increased with age in communities in Kilifi and Kwale counties, with higher seroprevalence in Jaribuni community than in other communities in Kilifi (*P* < 0.0001, Figure 3). Mean antibody responses against *P. malariae* MSP-1_19_ antigen also increased with age and were highest in Jaribuni (*P* < 0.0001), but very low in Ndau Island and Kipini communities (*P* < 0.0001 for difference with other communities).

**Figure 4.**
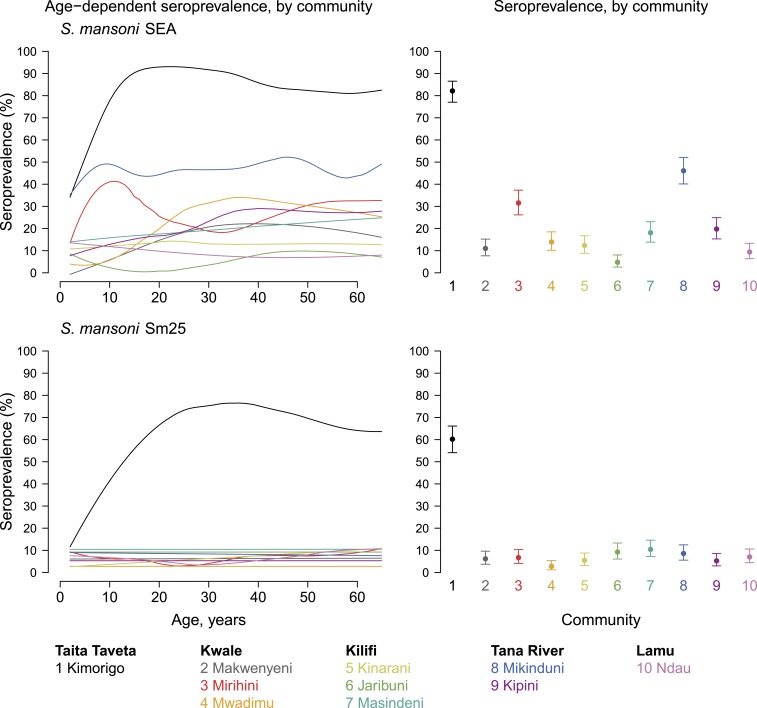
Schistosomiasis antibody age-dependent seroprevalence and overall means, stratified by community in Kenya’s coastal region, 2015. Community-level mean seroprevalence is age-adjusted and error bars represent 95% CIs. Supplemental Figure 4 is an extended version of this figure that also includes mean antibody levels. Created with script: https://osf.io/tpcg7. This figure appears in color at www.ajtmh.org.

Antibody responses against *S. mansoni* Sm25 recombinant antigen were primarily detected in Kimorigo community in Taita–Taveta County, and the seroprevalence increased gradually with age, reaching a peak at around 25 years of age (Figure 4). However, although antibody responses to *S. mansoni* SEA antigen also increased with age in Kimorigo community and mean seroprevalence was higher, there were some responses against this antigen in many other communities. Compared with the Sm25 recombinant antigen, the *S. mansoni* SEA antigen may have limited application in serosurveillance surveys because of cross-reactivity.

**Figure 5.**
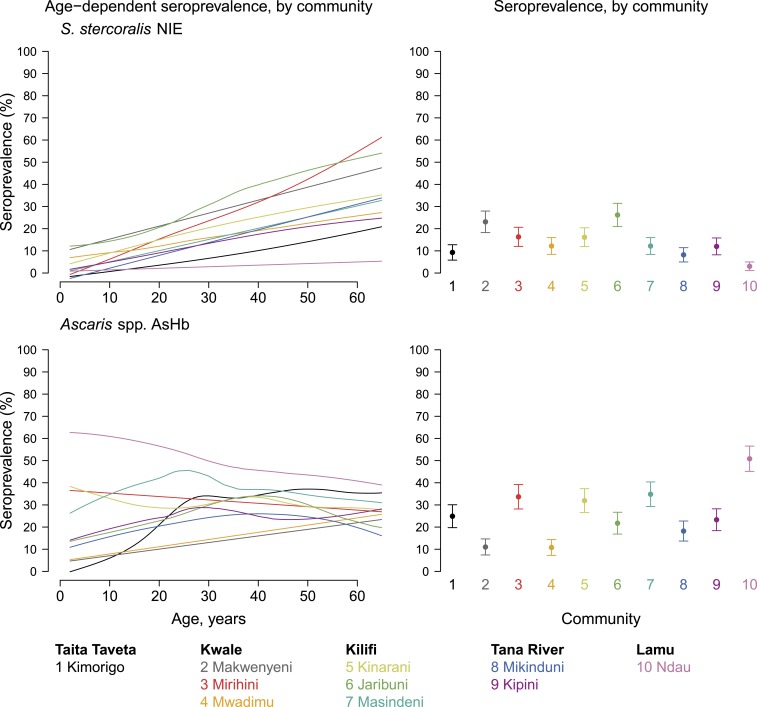
Age-dependent seroprevalence and overall mean for antibodies to *Strongyloides stercoralis* and *Ascaris* spp. stratified by community in Kenya’s coastal region, 2015. Community-level mean seroprevalence is age-adjusted and error bars represent 95% CIs. Supplemental Figure 5 is an extended version of this figure that also includes mean antibody levels. Created with script: https://osf.io/j7ux3. This figure appears in color at www.ajtmh.org.

Steady increases in *S. stercoralis* NIE seroprevalence with age were observed and community-level mean seroprevalence ranged between 3% and 26% (Figure 5). There was heterogeneity in age-dependent *Ascaris* Hb seroprevalence patterns across communities, with seroprevalence increasing with age in some communities and decreasing with age in others (Figure 5).

### Immune responses to vaccine-preventable diseases.

**Figure 6.**
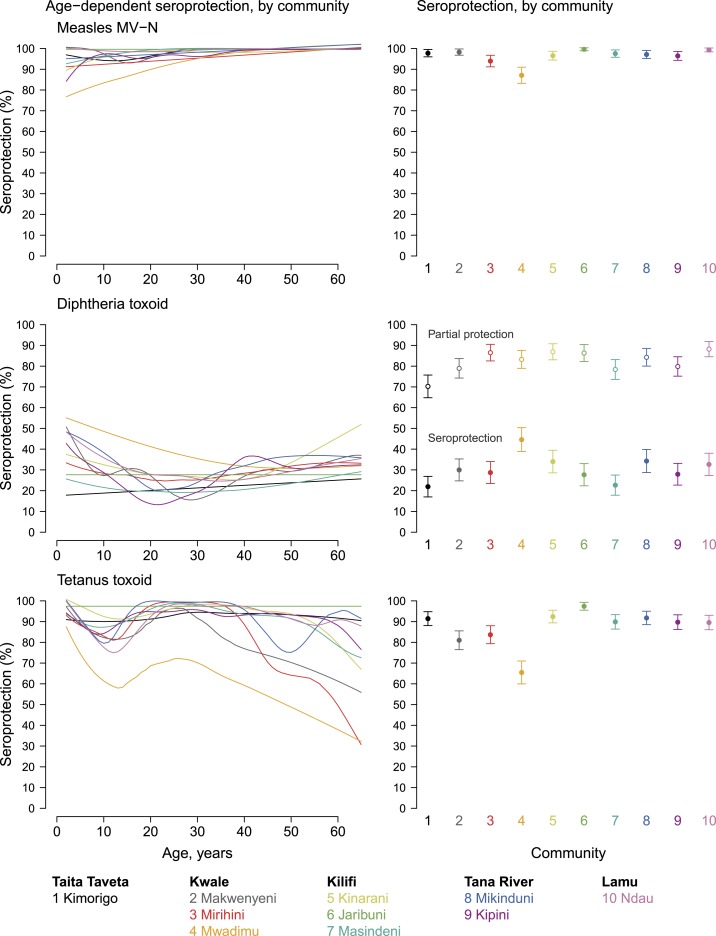
Age-dependent seroprotection and overall seroprotection for measles, diphtheria, and tetanus stratified by community in Kenya’s coastal region, 2015. Community-level seroprotection is age-adjusted and error bars represent 95% CIs. For diphtheria, we included separate community-level estimates of seroprotection (MFI-bg > 4,393 corresponding to 0.1 IU/mL) and partial protection (MFI-bg > 183 corresponding to 0.01 IU/mL). Supplemental Figure 6 is an extended version of this figure that also includes mean antibody levels. Created with script: https://osf.io/qrkhm. This figure appears in color at www.ajtmh.org.

Immune response against measles MV-N antigen increased with age, but two communities in Kwale County (Mirihini and Mwadimu) had < 90% seroprotection (Figure 6). Immune responses to diphtheria toxoid were relatively higher among children but waned slightly around the ages of 30–40 years before increasing slightly. Generally, diphtheria seroprotection ranged between 22% and 44% across communities, and partial protection (defined as responses of 0.01–0.099 IU/mL) ranged between 70% and 88% across communities. Immune responses against tetanus toxoid decreased by age in all communities until around 15 years when the levels increased again. However, tetanus seroprotection was lower in all three communities in Kwale County.

## DISCUSSION

Antibodies can provide valuable information about exposure to pathogens and can be helpful for characterizing transmission dynamics in an area to help prioritize where and what interventions may be considered.^24,45^ This study provides an important proof of concept for how the value of existing epidemiological surveillance activities could dramatically be increased using small volumes of blood collected on a filter paper and analyzed using a single multiplex laboratory assay and novel data analysis techniques.

The LF survey in the coastal region of Kenya, which provided the opportunity to collect information for this study, demonstrated that Ndau Island in Lamu County had the highest prevalence of CFA by ICT.^17^ The antifilarial antibody measurements assessed by the MBA platform closely aligned with the CFA results. Ndau Island had the highest levels of antibody responses to all three recombinant filarial antigens, which confirms the observation that LF transmission is presently higher in Ndau Island than in the other communities. Previous studies have demonstrated a spatial relationship between antibody-positive individuals and infected persons.^46^ The high seroprevalence rates in Ndau, especially among children, are consistent with the conclusion that transmission may be ongoing and not yet halted by the MDA campaign.

Antibody responses against Bm14 antigen continued to increase with age in all villages, which may have been an indication of cumulative exposure to *W. bancrofti*, and also likely reflects historic transmission. Generally, antibody responses against the three recombinant filarial antigens were higher among CFA-positive individuals than among CFA-negative persons although the difference was relatively smaller for Bm33 (see Supplemental Figure 7). Results from a recent study in American Samoa demonstrated that polymerase chain reaction-positive pools of LF vector mosquitoes were statistically significant predictors of seropositivity for Wb123 but not Bm14, suggesting Wb123 could be an indicator of ongoing transmission.^47^ Longitudinal studies in areas of intense LF transmission have shown that children acquire infections early in life.^48,49^ In addition, previous studies have demonstrated that antibody response against infective-stage filarial larvae antigen Wb123 is a specific measure of *W. bancrofti* infection, and reduction in both antibody prevalence and transmission is seen most clearly in young children.^50,51^ Quantitative antifilarial antibody responses among youngest children (aged 2–5 years and 6–10 years) provided much higher resolution distinctions between communities compared with seroprevalence using the same antigens or the ICT test (Supplemental Figure 8)—a result consistent with a recent analysis across diverse pathogens in low-transmission settings where seropositive individuals are rare.^18^ The higher resolution of quantitative antibody responses compared with seroprevalence, particularly when measured in small sampling clusters, suggests that quantitative antibody levels could serve as an important and more sensitive indicator of recent exposure in sentinel populations of young children and may be a valuable tool for surveillance in the context of LF elimination programs.^20^ Thus, combined measurement of these markers may be suitable for characterization of LF transmission settings, particularly toward the end of the program when the infection prevalence is very low.

There was high heterogeneity in malaria seroprevalence among the study communities, with Kwale and Kilifi counties generally showing relatively higher malaria transmission than the other three counties. The community mean seroprevalence values suggested that both *P. falciparum* and *P. malariae* transmission were highest in Jaribuni community in Kilifi County. These differences may reflect environmental heterogeneity in malaria larval breeding sites, and Jaribuni may be a hot spot for malaria transmission. A previous study in Kilifi and Kwale counties identified the primary vectors of malaria along the coast of Kenya to include *Anopheles funestus* and three members of the *An. gambiae* complex: *An. gambiae* s.s., *An. arabiensis*, and *An. merus*.^52^ The study also showed that relatively high malaria parasite prevalence can occur at low and even non-detectable levels of entomological inoculation rates (EIR), suggesting that measurement of EIR may be a relatively insensitive indicator of malaria transmission in some settings. Although malaria parasite prevalence and/or EIR have traditionally been used for reporting malaria transmission intensity,^53^ serological markers have increasingly been recognized as useful indicators for estimating malaria transmission intensity, which is key for assessing the impact of control interventions.^54–57^ Because of the longevity of the specific antibody response, seroprevalence reflects cumulative exposure and, thus, is less affected by seasonality or unstable transmission.^58^ ‬‬‬‬‬

In Kenya, *Schistosoma haematobium* is highly endemic along the coast where human exposure occurs primarily at pond and stream snail habitats.^9,59,60^ The absence of *S. mansoni* from most of the Kenyan coastal region is attributable to the absence of the *Biomphalaria* spp. intermediate-host snails.^61^ In Mikinduni community, along the lower Tana River, crude antigen SEA antibody responses were observed, but *S. mansoni*–specific Sm25 responses were lacking. By contrast, Taveta area in Taita–Taveta County is known to be endemic for both *S. haematobium* and *S. mansoni* infections,^62,63^ and this is reflected in the high SEA and Sm25 antibody responses we observed in Kimorigo, a community located on the banks of the shallow freshwater Lake Jipe. The absence of *S. mansoni* species–specific antibody responses to Sm25 recombinant antigen in all of the communities except Kimorigo confirms that *S. mansoni* infection is likely absent from the lower coastal areas. Thus, *S. mansoni* Sm25 recombinant antigen seems to be an excellent antigen for measuring antibody responses to *S. mansoni* infection,^64^ and SEA antigen likely detects antibody responses caused by both *Schistosoma* species by virtue of cross-reactivity.

The presence of responses to *S. stercoralis* NIE antigen is noteworthy because there has been little information on the geographic distribution of this helminth in Kenya due to diagnostic limitations. Copromicroscopic diagnostic methods commonly used in soil-transmitted helminthiasis prevalence studies are inadequate for *S. stercoralis* detection,^65^ and thus its distribution in many areas is unknown. Concentration methods, namely, the Baermann technique and Koga agar plate culture, have better but still unsatisfactory sensitivity.^66^ A study using NIE serology in Argentina found no cross-reactivity between *S. stercoralis* and infections with *A. lumbricoides*, hookworms, or *Hymenolepis nana*, and the presence of other helminths in the stool did not affect the *S. stercoralis*–specific antibody responses.^67^ A study comparing five serologic tests identified NIE–luciferase immunoprecipitation system to be the most accurate assay for the diagnosis of *S. stercoralis* infection.^68^ Previous studies using the recombinant NIE have documented high seroprevalence of *S. stercoralis* infection in remote Australian Indigenous communities and suggest that collection of DBSs may be a useful approach for field diagnosis of *S. stercoralis* seroprevalence.^69,70^ This study, therefore, provides evidence for possible low-level transmission of *S. stercoralis* in coastal Kenya as the seroprevalence varies from community to community. Community mean antibody responses to the *Ascaris* Hb native antigen and seroprevalence exhibited high heterogeneity among the study communities. A population-based study in Indonesia has shown that an assay for antibodies to *Ascaris* Hb is useful for assessing transmission of *Ascaris* infections, and community antibody rates decreased rapidly following MDA of anthelmintic drugs. The decrease was also found to reflect reduced egg excretion at the community level.^38^

Vaccination is one of the most cost-effective public health interventions available, and the epidemiology and burden of VPDs vary by country and by region partly because of differences in vaccine uptake.^71^ This multiplex-integrated serosurveillance study identified heterogeneity in serologic antibody levels against measles, diphtheria, and tetanus antigens. Our study demonstrates a need for regularly monitoring serological responses to vaccination programs in resource-poor settings where coverage may be low.

Some of the limitations of this study are somewhat similar to those highlighted previously.^24^ This was a preliminary, proof-of-concept study, and, because of the lack of direct comparison of results with those obtained from already validated commercial assays, the MBA described here was not intended to replace diagnostic tests that are presently available for use by disease control/elimination programs. Also, serological studies are traditionally faced with the challenge of establishing diagnostic cutoff points, especially when well-characterized positive and negative serum samples are not available. Nonetheless, the Gaussian mixture models applied in this study led to cutoff values that were very similar to those derived through ROC curves or from mean *plus* three SD calculations for malaria, LF, and helminth antibody responses (Figure 1). This result is consistent with a recent, multicountry comparison of cutoff methodology for trachoma antibodies^72^ and supports the use of finite mixture models to identify seropositivity cutoffs in studies without access to panels of known positive and negative specimens. For pathogens where cutoff values fall in the center of a unimodal distribution and it is more difficult to distinguish seropositive and seronegative groups (e.g., *A. suum* Hb in Figure 1), the use of community mean antibody levels avoids the requirement of choosing a cutoff, and observed antibody response patterns were very consistent with seroprevalence estimates across all of the antibodies tested in this study (Supplemental Figures 2–6). Another limitation of this study is potential for helminth antibody cross-reactivity, particularly because the MBA measured total IgG antibody rather than the more specific IgG4. Because the coastal area has a typical tropical climate, it is likely that a plethora of pathogens are coincident, some with potentially cross-reactive antigens. A previous study reported that cross-reactivity of the *Ascaris* Hb native antigen with hookworm and possibly *S. stercoralis* and *Toxocara* spp. limited its value in serology if one is interested in ascariasis alone.^38^ Thus, further studies are required to identify sensitive and specific recombinant antigens that could be used with more confidence in serological assays. A final limitation is that the multiplex assay we used in our study is not commercially available. In practice, it will be more realistic for programs to incorporate integrated antibody surveillance into their surveys when bead-based antibody assays can be purchased in a ready-to-use kit format, especially if the assays can be analyzed with instruments that do not rely on flow cytometry for fluorescent signal detection. Commercial kits with good quality control would potentially also help standardize the assays across laboratories.

Despite these limitations, this study successfully used a single multiplex-integrated serological assay and analysis methodology to measure antibody levels against several pathogens. There was no need to run separate assays for each pathogen, and we did not need to develop different mathematical models for each pathogen to compare exposure across communities and counties. The study highlighted overlap in pathogen burden that would not necessarily have been detected through single-disease surveillance. For example, Ndau Island was found to have not only the highest LF seroprevalence but also highest *Ascaris* seroprevalence, thus supporting integrated control of these two helminths. Interestingly, Ndau had almost no evidence for *P. falciparum* malaria transmission. On the other hand, Jaribuni community was found to stand out in terms of malaria, LF, and *Strongyloides*. Multiplex serosurveillance has the potential to enable us to look across diseases for opportunities for integrated control, thus providing synergy to global public health initiatives.

## CONCLUSION

This study highlighted the utility of the MBA platform for integrated serosurveillance of biomarkers of diseases of public health importance. The multiplex-integrated serologic assay has the potential to become an invaluable complementary epidemiologic tool for integrated monitoring of trends in endemicity of diseases of public health importance and the effectiveness of public health control programs.

## Supplemental figures

Supplemental materials
