# Supplementary material for: Integrated Cross-Sectional Multiplex Serosurveillance of IgG Antibody Responses to Parasitic Diseases and Vaccines in Coastal Kenya
Source: Am J Trop Med Hyg. 2019 Nov 25;102(1):164–76. doi: 10.4269/ajtmh.19-0365 (PMC6947807; doi:10.4269/ajtmh.19-0365)
Supplement: Supplementary file 1 [file tpmd190365.SD1.pdf]

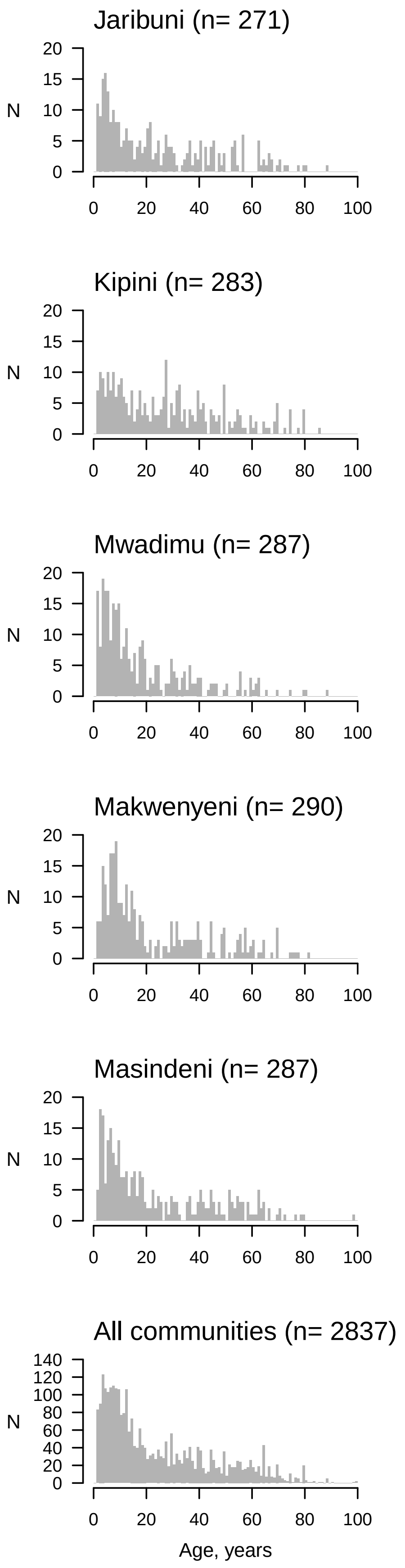

Figure S1 : Community level sample size and age distribution. Created with script: `https://osf.io/7jxmn`.

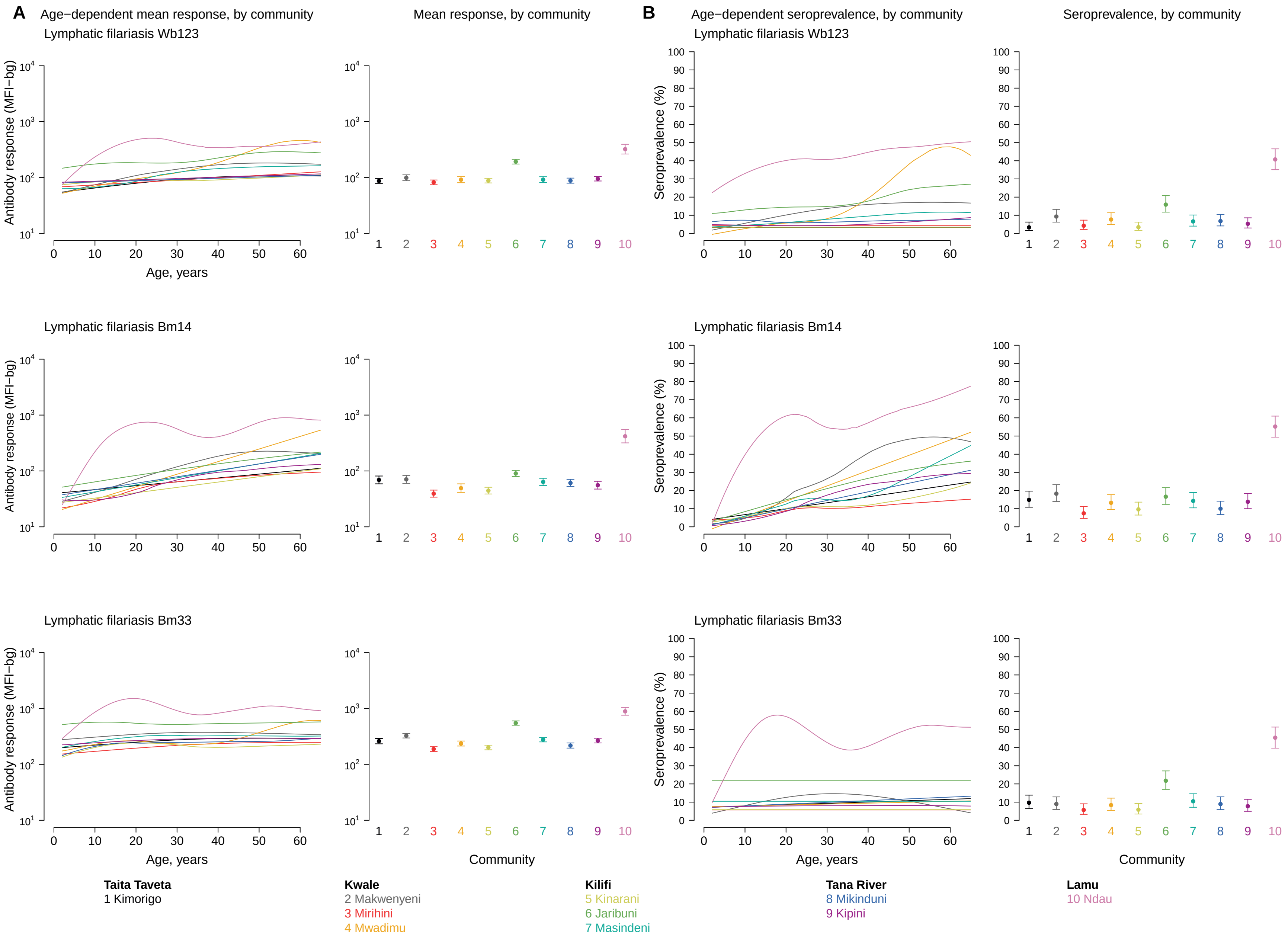

Figure S2 : Lymphatic filariasis antibody age-dependent mean response (**A**) and seroprevalence (**B**), stratified by community in Kenya's coastal region, 2015. Community-level mean antibody response and seroprevalence are age-adjusted and error bars represent 95% confidence intervals. Antibody response measured in median fluorescence units minus background (MFI-bg) on a BioRad Bio-Plex platform. Created with script: `https://osf.io/c79rw`.

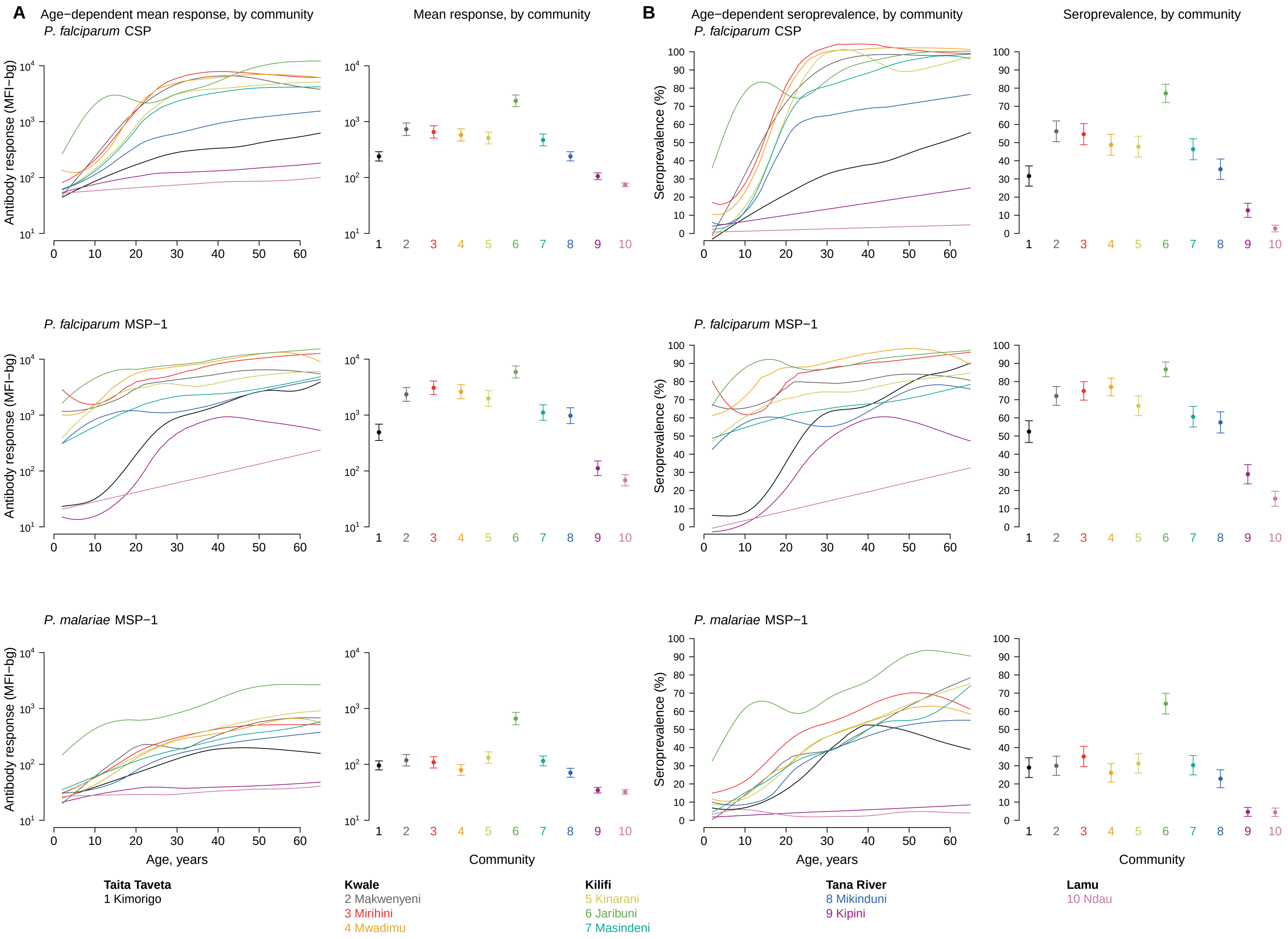

Figure S3 : Malarial antibody age-dependent mean response (**A**) and seroprevalence (**B**), stratified by community in Kenya's coastal region, 2015. Community-level mean antibody response and seroprevalence are age-adjusted and error bars represent 95% confidence intervals. Antibody response measured in median fluorescence units minus background (MFI-bg) on a BioRad Bio-Plex platform. Created with script: `https://osf.io/nhrc2`.

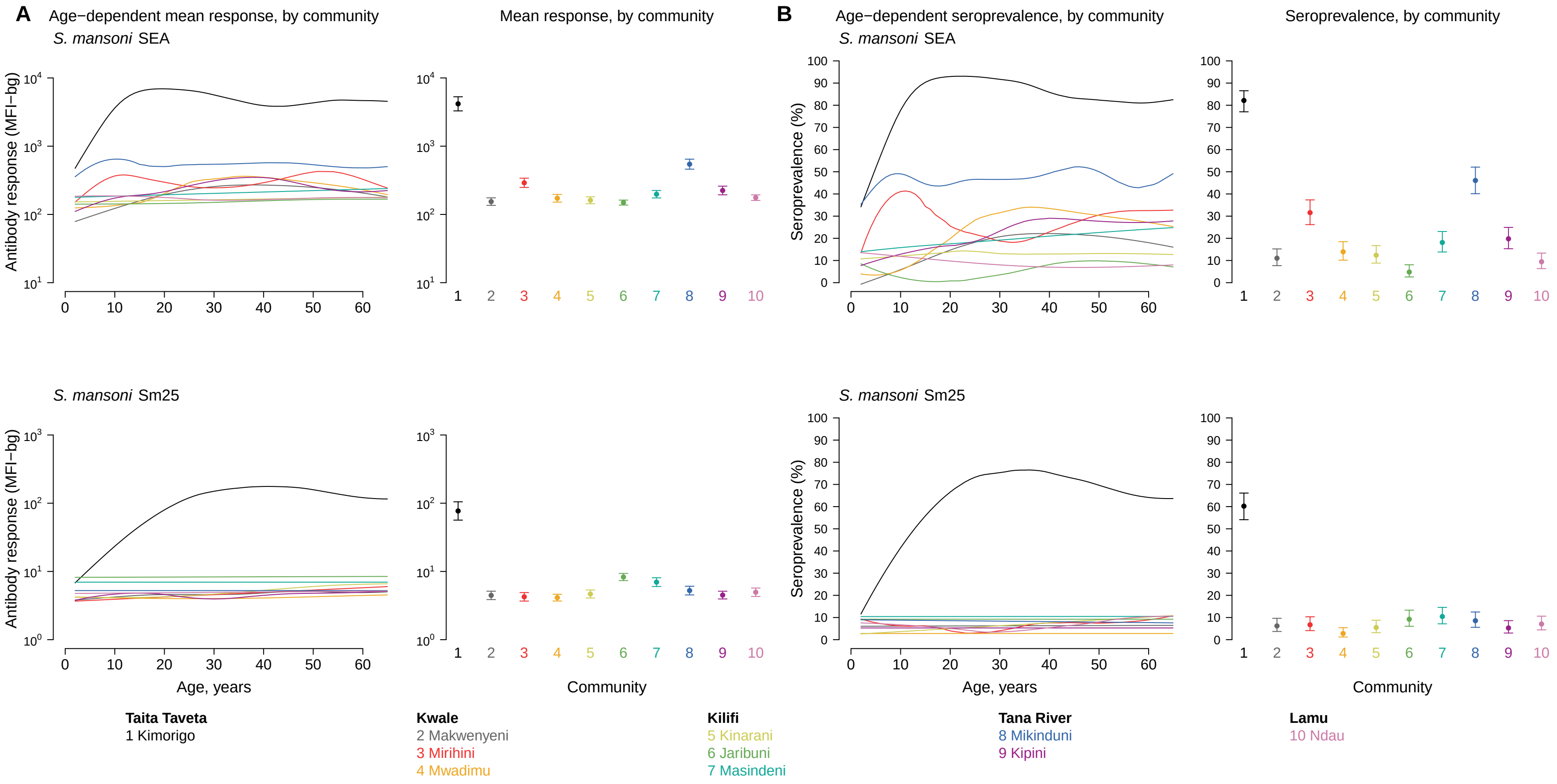

Figure S4 : Schistosomiasis antibody age-dependent mean response (**A**) and seroprevalence (**B**), stratified by community in Kenya's coastal region, 2015. Community-level mean antibody response and seroprevalence are age-adjusted and error bars represent 95% confidence intervals. Antibody response measured in median fluorescence units minus background (MFI-bg) on a BioRad Bio-Plex platform. Created with script: `https://osf.io/z8v4n`.

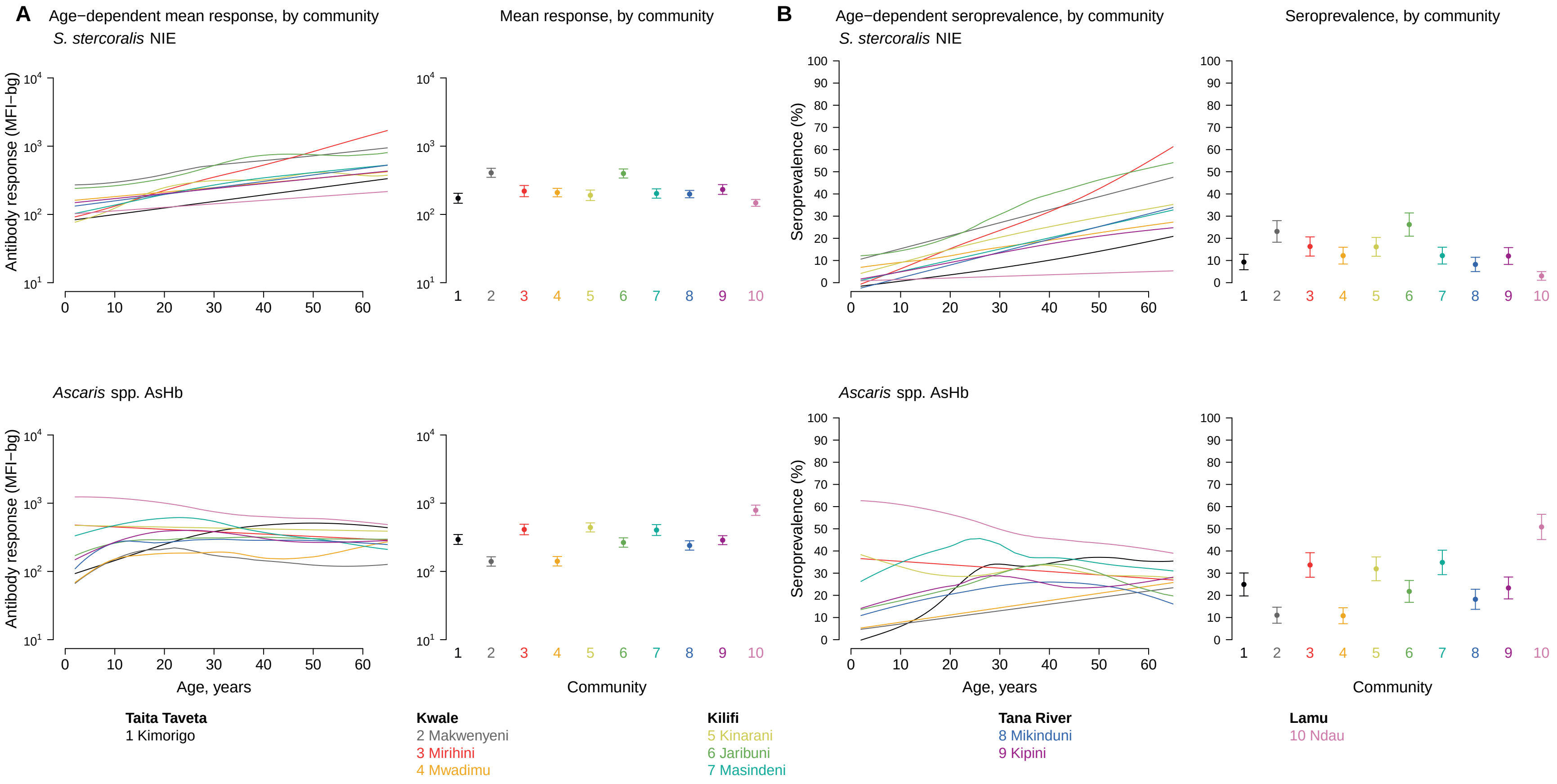

Figure S5 : Age-dependent mean response (**A**) and seroprevalence (**B**) to *S. stercoralis* and *Ascaris* spp. antigens, stratified by community in Kenya's coastal region, 2015. Community-level mean antibody response and seroprevalence are age-adjusted and error bars represent 95% confidence intervals. Antibody response measured in median fluorescence intensity minus background (MFI-bg) units on a BioRad Bio-Plex platform. Created with script: `https://osf.io/spnvx`.

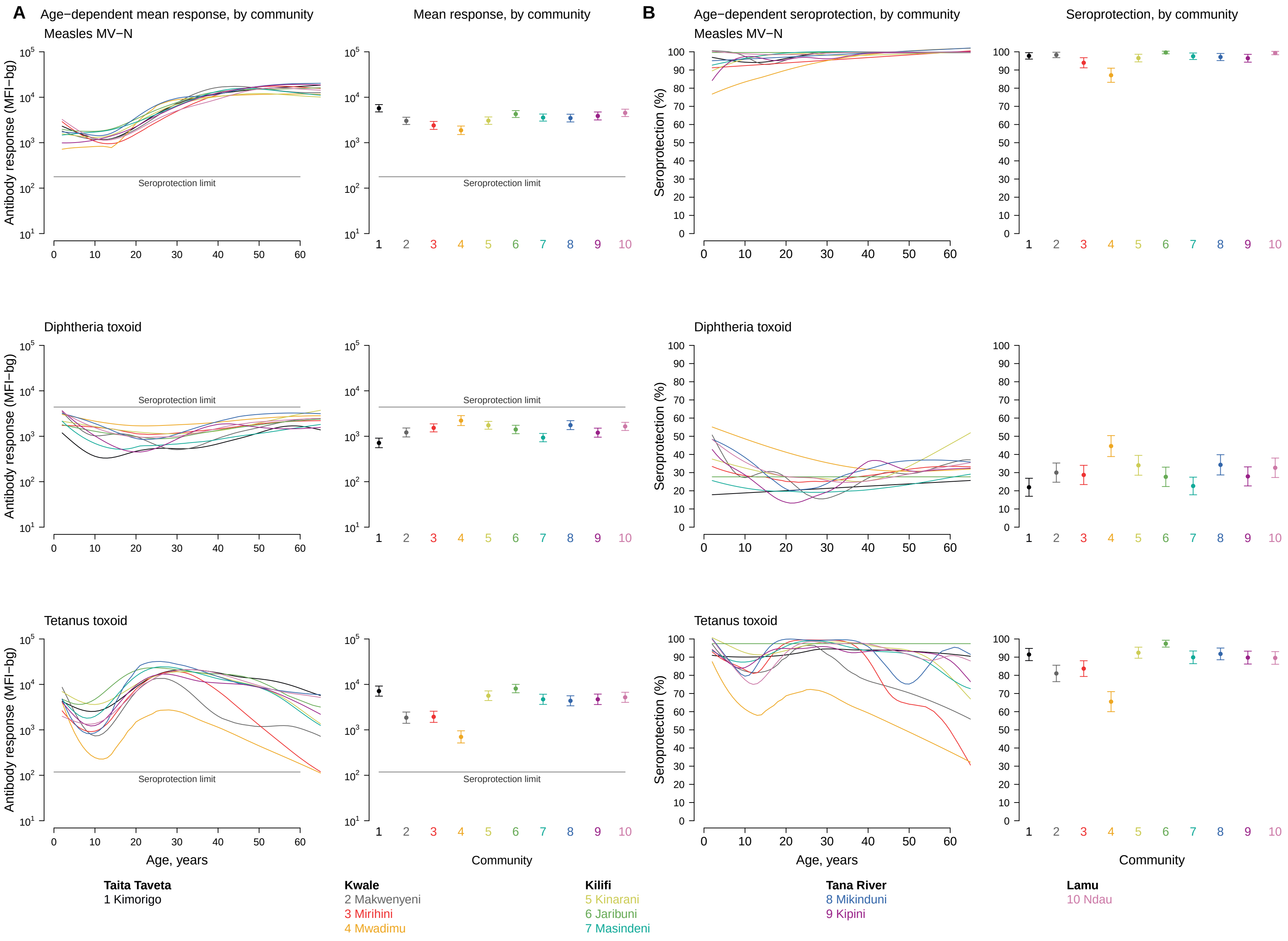

Figure S6 : Age-dependent mean response (**A**) and seroprotection (**B**) for measles, diphtheria, and tetanus stratified by community in Kenya's coastal region, 2015. Community-level mean antibody response and seroprotection are age-adjusted and error bars represent 95% confidence intervals. Antibody response measured in median fluorescence units minus background (MFI-bg) on a BioRad Bio-Plex platform. Created with script: `https://osf.io/uy5bf`.

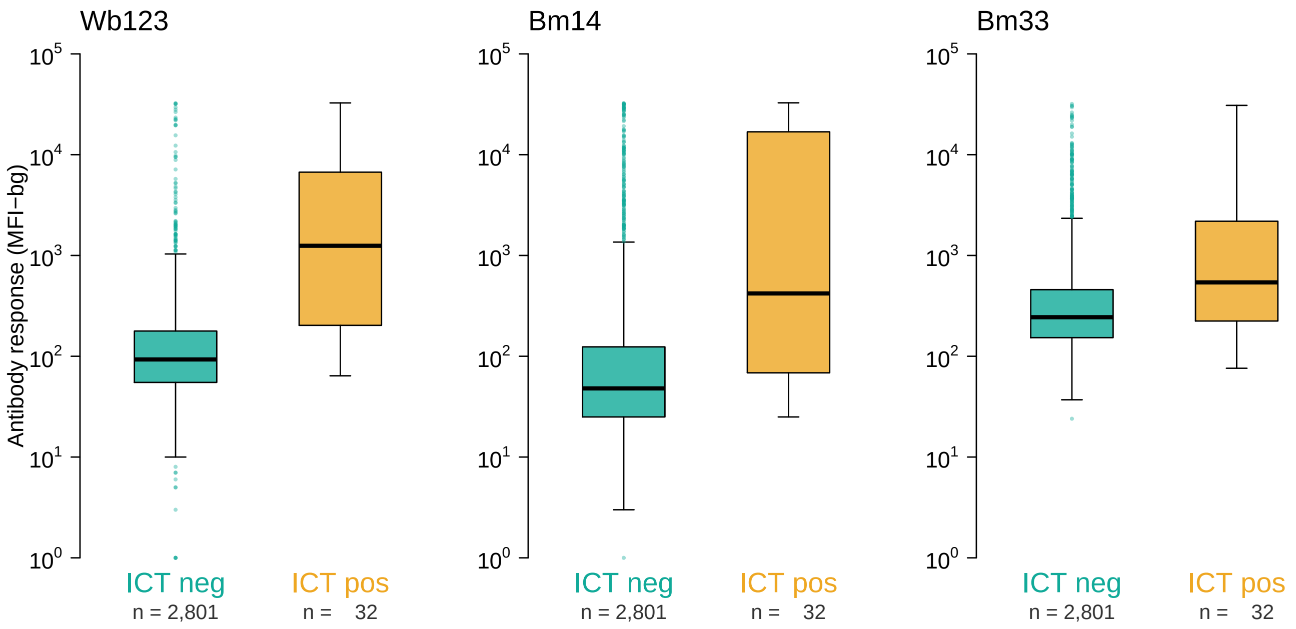

Figure S7 : Distribution of three lymphatic filariasis antibodies, stratified by rapid antigen immunochromatographic card test (ICT) results. Boxes mark the median and interquartile range of the distributions. Antibody response measured in median fluorescence units minus background (MFI-bg) on a BioRad Bio-Plex platform. Mann-Whitney U-test $P<0.0001$ for differences between ICT negative and positive individuals in antibody responses for each comparison (Wb123, Bm14, Bm33). Created with script: `https://osf.io/k9tms`.

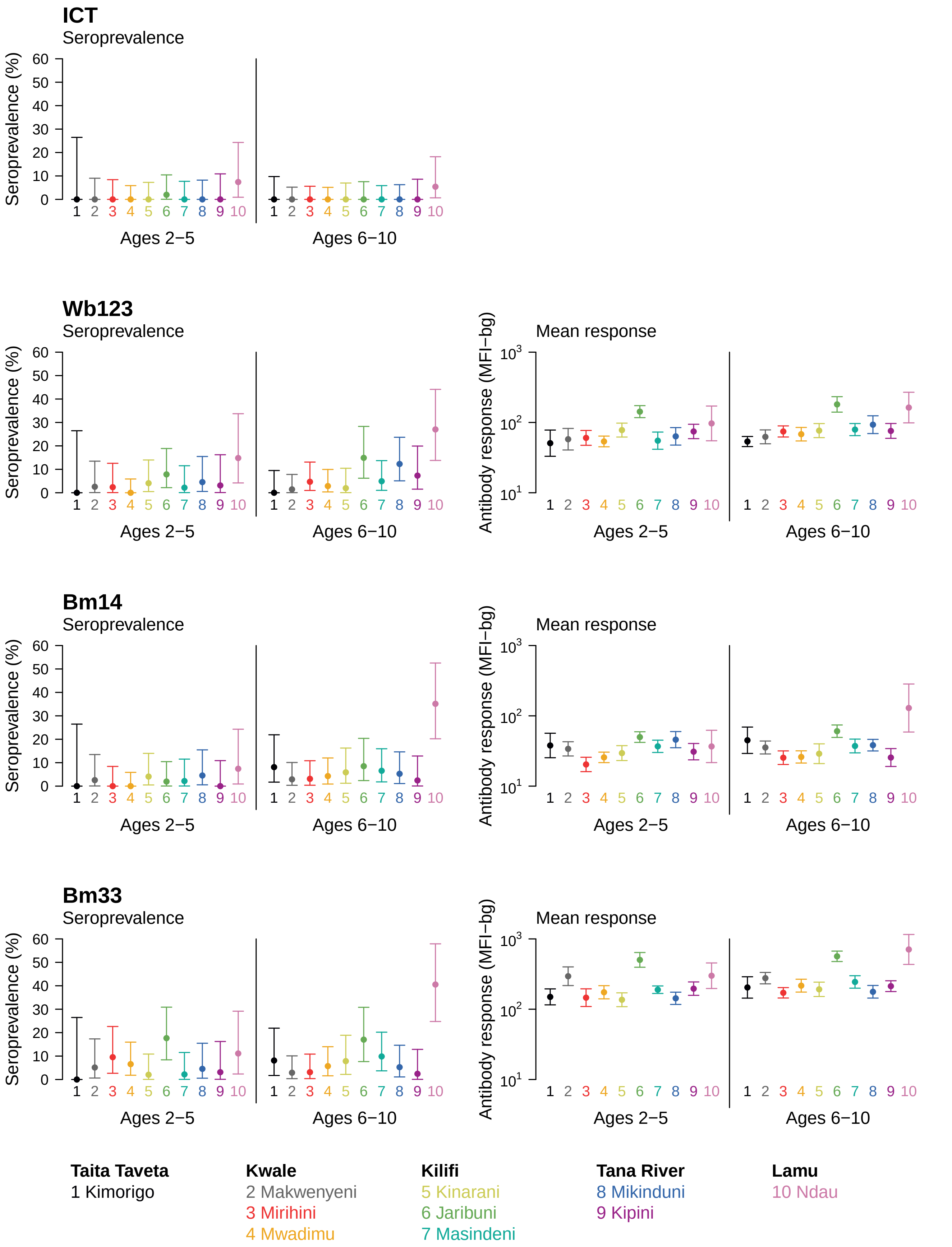

Figure S8 : Community level estimates of lymphatic filariasis seroprevalence and geometric mean antibody levels among children ages 2-5 and 6-10 years old. Child blood samples were tested the immunochromatographic card test (ICT) and three antigens (Wb123, Bm14, Bm33) measured in median fluorescence intensity minus background (MFI-bg) units on a multiplex BioRad Bio-Plex platform. The mean number of specimens tested per community within each age stratum was 47 (median=47; interquartile range=39, 58; range= 12, 70). Created with script: `https://osf.io/xh9yt`.
